# Percutaneous coronary intervention for a healed erosion with excimer laser coronary angioplasty and drug-coated balloon angioplasty: a case report

**DOI:** 10.3389/fcvm.2023.1153891

**Published:** 2023-08-21

**Authors:** Teruo Sekimoto, Shunya Sato, Hiroyoshi Mori, Hiroki Tanisawa, Hiroaki Tsujita, Seita Kondo, Hiroshi Suzuki, Toshiro Shinke

**Affiliations:** ^1^ Division of Cardiology, Department of Medicine, Showa University School of Medicine, Tokyo, Japan; ^2^ Division of Cardiology, Department of Medicine, Showa University Fujigaoka Hospital, Kanagawa, Japan

**Keywords:** acute coronary syndrome, optical coherence tomography, intravascular ultrasound, healed plaque, excimer laser, drug-coated balloon

## Abstract

**Background:**

Healed plaque, characterized by distinct layers of organizing thrombus and collagen, is the hallmark of tissue self-repair. However, the efficacy of excimer laser coronary angioplasty (ELCA) followed by drug-coated balloon (DCB) angioplasty in patients with healed plaques is not fully understood.

**Case summary:**

A 42-year-old woman with a history of anxiety disorder was admitted to our institution with worsening chest pain and subsequently diagnosed with anterior non-ST-elevation myocardial infarction. Coronary angiography revealed severe stenosis in the proximal left anterior descending artery (LAD) despite Thrombolysis in Myocardial Infarction (TIMI) grade 3. Optical coherence tomography (OCT) showed healed plaques with partial macrophage accumulation and no fresh thrombus. Plaque disruption and thin-cap fibrous atheroma were not identified in the culprit lesions. Intravascular ultrasound (IVUS) confirmed high-intensity marginal irregular masses at the culprit site, suggesting that the thrombus was formed by plaque erosion rather than lipid plaque or necrotic tissue. With lesion modification using ELCA prior to DCB angioplasty, OCT examination of the LAD after ELCA showed a significant reduction in plaque burden and preserved lumen size. Post-percutaneous coronary intervention angiography revealed no stenosis with TIMI grade 3. A follow-up coronary computed tomography scan showed no angiographic restenosis, and the patient remained symptom-free.

**Conclusions:**

Here we describe a case in which OCT and IVUS evaluation suggested organizing thrombus due to erosion healing, and a favorable outcome was achieved with the combination of ELCA and DCB. The combination use of ELCA and DCB might be a potential strategy for acute coronary syndrome patients with organizing thrombus.

## Introduction

Acute coronary syndrome (ACS) is primarily caused by plaque rupture or erosion with superimposed occlusive thrombosis. However, pathological studies have suggested that some plaques may rupture silently without causing symptoms, subsequently leading to healed plaques (1). Optical coherence tomography (OCT) is a high-resolution intracoronary imaging modality that allows the identification of the etiology of ACS and healed plaques as described in histological examinations (2, 3). On the other hand, excimer laser coronary angioplasty (ELCA) has been demonstrated safe and effective in acute ischemic-thrombotic coronary syndromes by the successful vaporization of intracoronary thrombus at the target lesion (4). However, the efficacy of ELCA in patients with healed plaques is not yet fully understood. Here we report a case of ACS suspected to feature healed erosions by assessing OCT and intravascular ultrasound (IVUS) in a patient who was treated with ELCA without stenting.

## Case description and diagnostic assessment

A 42-year-old woman with worsening chest pain was admitted to our institution. She had become aware of chest discomfort with stress and at dawn for 1 month before her visit, but the symptoms had increased in frequency without any triggers. She was a non-smoker, and her medical history included anxiety disorder controlled by medication. On admission, the blood pressure was 125/69 mmHg, heart rate was 66 bpm, and oxygen saturation was 98% on room air. A 12-lead electrocardiogram showed a new negative T wave in V1–4. On arrival, her troponin I levels were 181.3 pg/ml (normal range, <15.6 pg/ml). Her low-density lipoprotein level was 135 mg/dl (upper limit of normal, 140 mg/dl); high-density lipoprotein level, 64 mg/dl (lower limit of normal, 35 mg/dl); triglyceride level, 128 mg/dl (upper limit of normal, 150 mg/dl); and glycated hemoglobin level, 5.3% (upper limit of normal, 6.2%). Chest radiography revealed a normal-sized heart with no lung consolidation or abnormalities. Transthoracic echocardiography showed mild hypokinesis of the ante-septal wall with preserved left ventricular systolic function and an ejection fraction of 60%.

**Figure 1:**
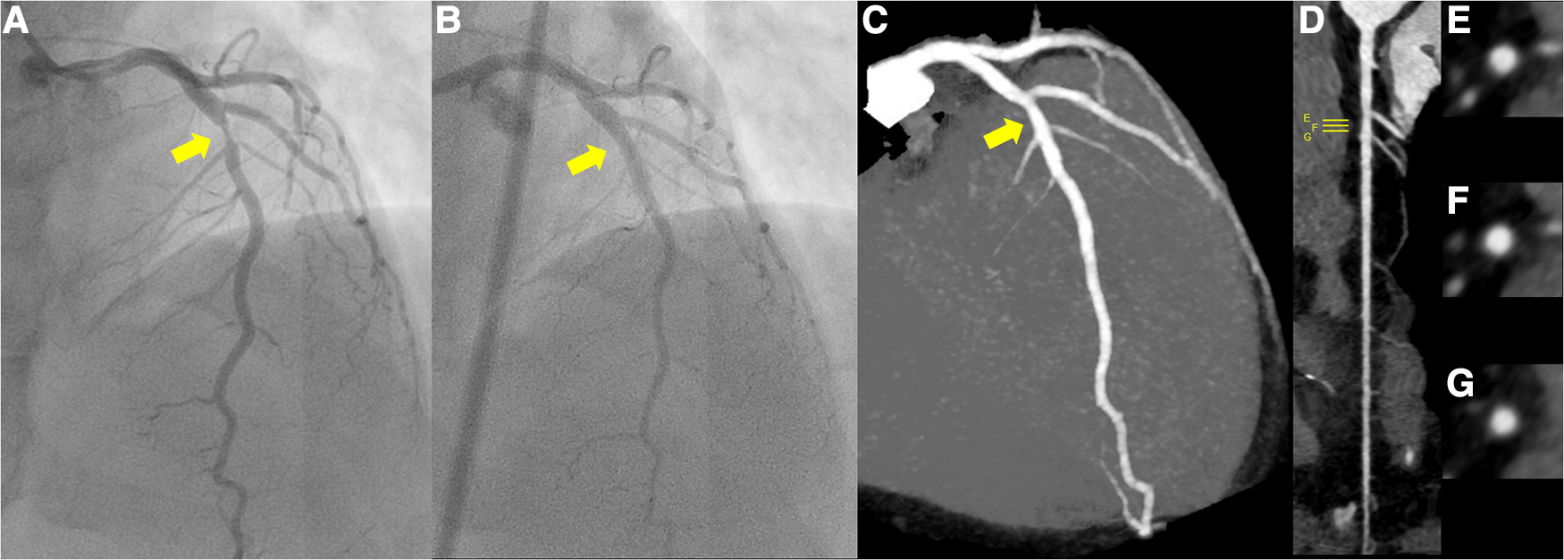
(**A**) Pre-percutaneous coronary angiogram (PCI) revealing severe stenosis of the left anterior descending coronary artery (yellow arrow). (**B**) Post-PCI coronary angiogram revealing significant reduction in stenosis of the culprit lesion (yellow arrow). (**C–G**) Follow-up coronary computed tomography scan at 1 year showed a small soft eccentric plaque with very mild positive remodeling and less than 20% luminal stenosis (yellow arrow; **C**, maximum intensity projection; **D,** stretched-curved planar reconstruction; **E–G**, cross-sections).

**Figure 2:**
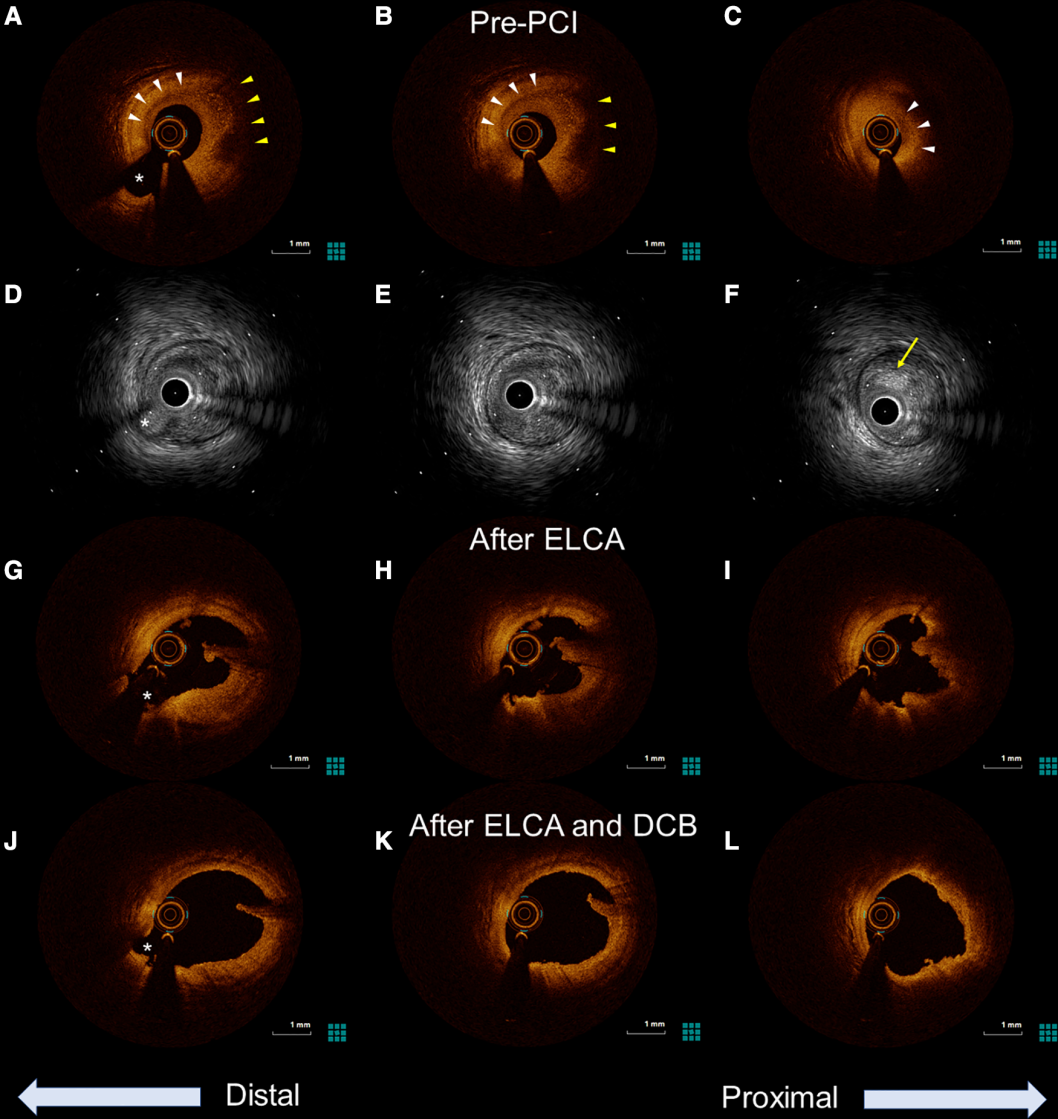
Optical coherence tomography (OCT) and intravascular ultrasound (IVUS) images of the culprit lesion from distal to proximal. The asterisk (*) marks the second diagonal branch as a landmark. (**A–C**) Pre-percutaneous coronary angiogram (PCI) OCT revealing the presence of healed plaque (white arrowhead). The partial accumulation of macrophages was also identified in the healed plaque (yellow arrowhead). Minimum lumen area was 0.87 mm^2^. (**D–F**) Pre-PCI IVUS image showing a high-intensity marginal irregular mass in the culprit site (yellow arrow), suggesting thrombus rather than lipid plaque or necrotic tissue. (**G–I**) OCT examination in the LAD after excimer laser coronary angioplasty showing significant thrombus reduction and a preserved lumen size. (**J,K**) Further lumen expansion was achieved with the addition of balloon dilatation, and the minimum lumen area expanded to 5.49 mm^2^.

The patient was loaded with 200 mg of aspirin and immediately taken to the cardiac catheterization laboratory. Emergency coronary angiography revealed severe stenosis proximal to the left anterior descending artery (LAD) despite the intracoronary injection of nitroglycerin (Figure 1A), which was determined to be the culprit lesion. Immediate percutaneous coronary intervention (PCI) was indicated, for which 20 mg of prasugrel was administered. OCT of the LAD was performed to evaluate the underlying etiology of the ACS. The OCT (Dragonfly™ OPTIS™ and ILUMIEN™ OPTIS-System™; Abbott Vascular, St. Paul, MN, USA) revealed the presence of healed plaque (white arrowhead) with partial accumulation of macrophages (yellow arrowhead), and the minimum lumen area was 0.87 mm2 (Figures 2A–C). Plaque disruption and thin-cap fibrous atheroma were not identified in the culprit lesions. In contrast, IVUS (AltaView^TM^; Terumo, Tokyo, Japan) confirmed high-intensity marginal irregular masses in the culprit site, suggesting thrombus formation by plaque erosion rather than lipid plaque or necrotic tissue (Figures 2D–F). Considering that the patient was a young premenopausal woman, we decided to proceed with lesion modification with ELCA and complete the procedure with balloon dilation without stenting to shorten the antiplatelet therapy period. ELCA was subsequently performed using a pulsed xenon-chloride excimer laser system with a wavelength of 308 nm; pulse duration of 135 ns; and output of 175 mJ/pulse (CVX-300P; Royal Philips, Amsterdam, Netherlands). A 0.9-mm concentric ELCA catheter (ELCA™ Coronary Laser Atherectomy Catheter; Royal Philips) was advanced slowly at a speed of 0.5 mm/s through the lesion while excimer laser was delivered. We started with laser energies at a fluence of 30 mJ/mm² and a repetition rate of 25 Hz. The laser energy was then increased to 80 mJ/mm² with a repetition rate of 80 Hz for three additional sequences. OCT examination of the LAD after ELCA showed a significant reduction in plaque burden and preserved lumen size (Figures 2G–I). The irregularity of the endoluminal surface after ELCA suggested that some of the plaque containing thrombus was partially evaporated by the laser, exposing the underlying thrombus. To achieve optimal lesion preparation and prevent dissection progression prior to dilation with drug-coated balloon (DCB), a 3.00 × 10 mm cutting balloon (Wolverine; Boston Scientific, Natick, MA, USA) was utilized for pre-dilatation up to 8 atm pressure. Subsequently, a 3.0 × 20 mm DCB (SeQuent Please^TM^, B. Braun Melsungen AG, Berlin, Germany) was used for 30 s at 8 atm. Final OCT images (Figures 2J–L**)** revealed favorable results, with the minimum lumen area expanding to 5.49 mm². Post-PCI angiography demonstrated no stenosis, with a TIMI grade 3 flow (Figure 1B).

The peak troponin I levels were 1,535.1 pg/ml. Based on her mild bleeding risk (HAS-BLED score of 1, PRECISE-DAPT score of 9) and the absence of stent implantation, the patient received dual antiplatelet therapy with 100 mg of aspirin and 3.75 mg of prasugrel for 12 weeks, followed by continuation of prasugrel as a single antiplatelet therapy. Rosuvastatin 10 mg was also added for secondary prevention of ACS. As the patient's chest pain with stress and at dawn suggested the association of coronary vasospasm in the development of plaque erosion, calcium channel blockers (CCBs) were administered. A 1-year follow-up coronary computed tomography scan showed a small soft eccentric plaque with very mild positive remodeling and less than 20% luminal stenosis (Figures 1C–G), and the patient remained symptom-free (The timeline was summarized in Table 1).

**Table 1:** Timeline.

Presentation	• The patient was admitted to our institution with worsening chest pain. • Electrocardiography and laboratory data showed non-ST-elevation myocardial infarction.
Cath lab	• Emergent coronary angiography showed 90% stenosis in the proximal left anterior descending artery (LAD) with Thrombolysis in Myocardial Infarction grade 3. • Optical coherence tomography and intravascular ultrasound of the LAD revealed healed plaque with organizing thrombus. • Excimer laser coronary angioplasty was performed in the proximal LAD. • The procedure was completed with balloon dilation without stenting.
Day 2	Dual antiplatelet therapy with prasugrel and aspirin and calcium channel blockers were initiated.
12 weeks	Prasugrel as a single antiplatelet therapy was continued.
One-year	• The patient remained symptom-free. • A follow-up coronary computed tomography scan at 1 year showed no angiographic restenosis.

## Discussion

Healed plaques are recognized by their different optical features in the different layers. Fresh thrombi undergo complex processes, including granulation tissue formation with proteoglycans, Type III collagen deposition, smooth muscle cell proliferation, and re-endothelization (5). This healing process results in new tissue layer formation. A validation study in which healed plaques were identified by OCT as plaques with one or more layers of different optical densities reported high sensitivity and specificity for the *in vivo* identification of layered plaque patterns by histopathology (3). Interestingly, the findings of suspected organizing thrombus in the proximal culprit site were quite different between OCT and IVUS. This can be explained by the signal penetration depth of each imaging modality. The low penetration depth of OCT (1.5–2 mm) limited the detection of the organizing thrombi. In contrast to OCT, IVUS has a deep signal penetration (approximately 5 mm), which may allow its visualization. Therefore, depending on the healing process of the organizing thrombus, healed plaques may not be detectable using OCT alone.

Despite the development of second-generation drug-eluting stents (DES), challenges remain with respect to complications such as in-stent restenosis and stent thrombosis. A study by Merinopoulos et al. in 1,139 STEMI patients compared the outcomes of *de novo* lesions treated with DCBs and second-generation DES (6). The results showed no significant difference in all-cause mortality over a median follow-up of 3 years. A randomized trial demonstrated non-inferiority of the DCB strategy compared to DES implantation in ACS patients with residual stenosis <50% (7). ELCA is particularly effective at debulking and ablating both plaque and thrombus (8). The reduction of intracoronary thrombus in the culprit lesion may play a crucial role not only in reducing complications such as distal embolization and no-reflow phenomenon, but also as lesion preparation for DCB. Therefore, the combined use of ELCA and DCB may be a strategy for patients with ACS with an organizing thrombus. However, in a study evaluating the prognosis of patients with plaque erosion treated with antiplatelet therapy without stent implantation, significant area stenosis and high thrombus burden were found to be risk factors for cardiac events (9). Therefore, it is important to monitor plaque progression based on area stenosis and thrombus volume before PCI.

Plaque erosion is reportedly associated with vasospasm. An OCT study evaluated morphological characteristics of coronary artery spasm sites in 69 patients with vasospastic angina and demonstrated that thrombi were frequently (28%) detected at coronary artery spasm sites, while plaque erosion was common in patients with vasospastic angina (26%) (10). A recent study analyzing 39 patients with suspected vasospastic angina and an organic lesion using OCT reported that organic lesions related to coronary artery spasm were frequently accompanied by healed plaques and macrophage infiltration (93% and 80%, respectively) (11). In addition to the typical symptoms of vasospastic angina, the OCT findings were consistent with those of previous studies suggesting plaque erosion with a vasospastic background. CCBs are recommended as first-line treatment for newly diagnosed vasospastic angina (12) and may have contributed to symptom relief after PCI. Although we considered performing a spasm provocation test, we could not obtain her consent. Identification of vasospasm assessed by spasm provocation test might help to understand not only ACS etiology but also optimize medical therapy, including the addition of calcium channel blockers.

## Data Availability

The raw data supporting the conclusions of this article will be made available by the authors, without undue reservation.
